# PANDA-3D: protein function prediction based on AlphaFold models

**DOI:** 10.1093/nargab/lqae094

**Published:** 2024-08-06

**Authors:** Chenguang Zhao, Tong Liu, Zheng Wang

**Affiliations:** Computer and Information Sciences Department, St. Ambrose University, 518 W Locust St, Davenport, IA 52803, USA; Department of Computer Science, University of Miami, 1365 Memorial Drive, Coral Gables, FL 33124, USA; Department of Computer Science, University of Miami, 1365 Memorial Drive, Coral Gables, FL 33124, USA

## Abstract

Previous protein function predictors primarily make predictions from amino acid sequences instead of tertiary structures because of the limited number of experimentally determined structures and the unsatisfying qualities of predicted structures. AlphaFold recently achieved promising performances when predicting protein tertiary structures, and the AlphaFold protein structure database (AlphaFold DB) is fast-expanding. Therefore, we aimed to develop a deep-learning tool that is specifically trained with AlphaFold models and predict GO terms from AlphaFold models. We developed an advanced learning architecture by combining geometric vector perceptron graph neural networks and variant transformer decoder layers for multi-label classification. PANDA-3D predicts gene ontology (GO) terms from the predicted structures of AlphaFold and the embeddings of amino acid sequences based on a large language model. Our method significantly outperformed a state-of-the-art deep-learning method that was trained with experimentally determined tertiary structures, and either outperformed or was comparable with several other language-model-based state-of-the-art methods with amino acid sequences as input. PANDA-3D is tailored to AlphaFold models, and the AlphaFold DB currently contains over 200 million predicted protein structures (as of May 1st, 2023), making PANDA-3D a useful tool that can accurately annotate the functions of a large number of proteins. PANDA-3D can be freely accessed as a web server from http://dna.cs.miami.edu/PANDA-3D/ and as a repository from https://github.com/zwang-bioinformatics/PANDA-3D.

## Introduction

Proteins, the essential functional units of life, play crucial roles in catalyzing biochemical reactions (1), providing structural support for anaphase spindle (2), and regulating gene expressions (3,4). Accurately annotating protein functions is important for understanding biological processes and discovering novel drug targets (5,6). However, experimentally determining the functions of proteins is both laborious and expensive (7), whereas machine learning approaches can decrease the time and cost required for this task making accurate and comprehensive annotations possible and offering a promising avenue for protein function prediction.

Amino acid sequences determine protein structures (8,9), and protein structures determine the function of proteins (10). Therefore, proteins that share similar sequences may have similar functions. For a considerable length of time, protein function predictors focus on using machine learning methods to leverage the sequence alignment, as revealed by the critical assessment of functional annotation (CAFA) challenge. The keyword analyses of the top ten methods in CAFA2 (11) and of all participating methods in CAFA3 (7) show that sequence alignment and machine learning are the two most frequently used approaches. Our previous tool PANDA (12) uses profile-profile alignments and PSI-BLAST (13) to find similar proteins, detects reserved protein domains, executes a Bayesian model to infer GO terms from domain architectures, and then combines the candidate GO terms from these approaches to make final predictions. DeepGOPlus (14) uses a one-dimensional (1D) convolutional neural network (CNN) to predict the protein functions from amino acid sequences. GODoc (15) applies a *k*-nearest-neighbor algorithm over sequence information, such as amino acid-coupling pattern representations, to predict protein functions. DEEPred (16) makes predictions by feeding the sequence features, such as subsequence profile map and pseudo amino acid composition, into stacked feed-forward deep neural networks (DNN) followed by a hierarchical post-processing method. ProLanGO (17) used a recurrent neural network (RNN)-based machine translation model to predict protein functions from protein sequences.

In addition to extracting knowledge from protein sequence alignment, some newer methods leverage that by using protein language models in the last few years. Our PANDA2 (18) utilizes a graph neural network (GNN) to model the GO-directed acyclic graph (GO-DAG) topology and incorporates features generated by the protein large language model (LLM) (19). UDSMProt (20) uses a self-supervised RNN to learn task-agnostic representations of sequences, which is then fine-tuned on the downstream task of protein function prediction. Littmann *et al.* (21) found that predicting GO terms based on proximity of embeddings from language models SeqVec (22) or ProtBert (23) outperformed naïve sequence-based transfer. Rives *et al.* (19) trained a deep transformer (24) on about 250 million sequences, and the embedding generated by this evolutionary-scale language modeling (ESM) contains information on protein structures, functions, and binding information, which outperformed others in a variety of downstream tasks (19). This pre-trained language model was used by many state-of-the-art methods, such as ATGO (25), DeepGO-SE(26), SPROF-GO (27) and NETGO3 (28).

Following the recent successful prediction of the protein three-dimensional (3D) structures, a few methods shifted the focus towards integrating sequence alignment, protein structure, and machine learning. DeepFRI (29) applies a graph convolutional network (GCN) to the features generated by a protein language model and experimentally determined structures. Because the DeepFRI model was trained on experimentally determined structures, the performance on predicted models was worse than that on experimentally determined structures (29). Also, DeepFRI does not fully capture the 3D information because the input tertiary structure is first converted to a 2D contact map before being fed into the GCN architecture. Similarly, GAT-GO (30) also converts the input tertiary structure to inter-residue contacts first and then feeds the 2D contact map into the architecture. COFACTOR predicts GO terms by combining independent predictions from structure-based (31), sequence-based, and protein-protein-interaction-based pipelines (32).

Advanced learning architectures have been developed to extract knowledge from protein tertiary structures for research topics including protein sequence design (33,34), generative models of proteins (35) and inverse folding (36). When designing PANDA-3D, we believed and later verified that these learning architectures can comprehensively and efficiently capture knowledge from protein 3D structures. Usually, two formats of features can be extracted from protein 3D structures: vector features and scalar features. Vector features can be the orientation of residues in the protein structure, whereas scalar features can be distances and angles. The popular GNNs, such as Battaglia *et al.* (37), usually cannot operate on the scalar and vector features simultaneously. The geometric vector perceptron (GVP) was specially designed for learning 3D macromolecular structures with scalar channels and vector channels (38,39). GVPs have been proven to have advantages over convolutional neural networks and graph neural networks for model quality assessment and computational protein design (39). Hsu *et al.* (36) revealed that the geometric reasoning capability of GVP-GNN layers is complementary to transformer layers for inverse folding. Therefore, we incorporated GVP-GNN in the learning network of PANDA-3D, and the output from the GVP-GNN is fed into a transformer decoder.

The transformer model (40) has shown great success in a wide range of tasks, such as natural language processing (24) and vision tasks (41). However, some protein function predictors utilize only the encoder blocks in their architectures (42,43) since the decoder block was not originally designed for multi-label classifications. We feed all GO terms used for prediction into the transformer decoder so that the decoder can learn the relationships or co-occurrence patterns among all possible GO terms, which are then combined with the output from the encoder by cross-attentions.

## Materials and methods

### PANDA-3D architecture

**Figure 1.**
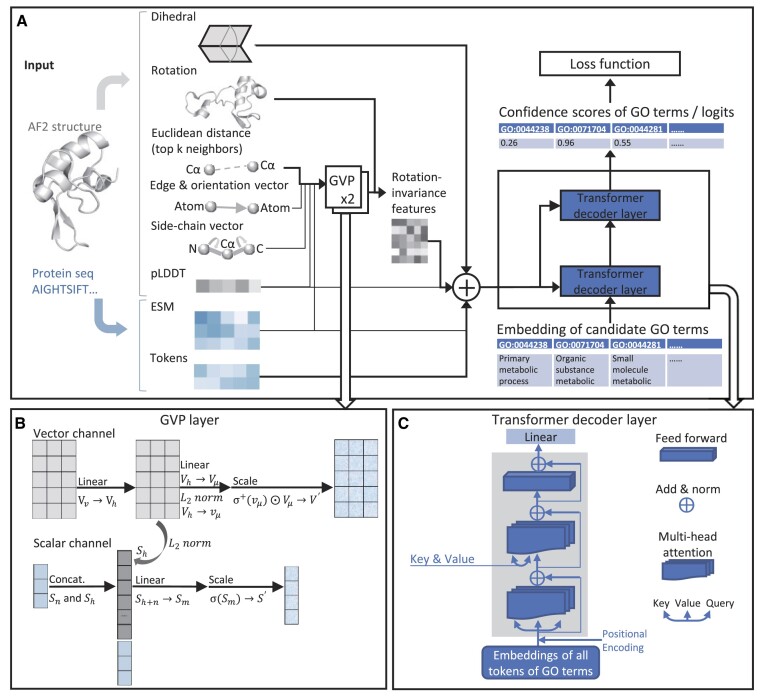
The overall architecture of PANDA-3D. Panel **A** shows that the GVP-GNN are used to extract information from predicted structures and protein sequence embeddings, succeeded by a variant transformer decoder producing confidence scores over query GO terms. Panel **B** shows the scalar and vector channels of a GVP layer. Panel **C** shows the architecture of a transformer decoder layers for multi-label classification.

The architecture of PANDA-3D is depicted in Figure 1. We developed the encoder of PANDA-3D by modifying the GVP-transformer encoder blocks proposed for inverse folding (36). This modified encoder utilizes the geometric reasoning capability of GVP-GNN layers (39). In addition, we implemented the decoder layers specialized for multi-label classification inspired by (44). We fed all PANDA-3D candidate classes, which are GO terms, as the query into the decoder, allowing PANDA-3D to compute self-attention for the GO terms. The updated query obtained from the self-attention layer was subsequently utilized to calculate cross-attention with the output from the encoder layers. This enables PANDA-3D to learn the relationships between GO terms and the structure and sequence features through the cross-attention layer.

#### Features

##### Structure-based features fed into GVP layers

To generate embeddings from AlphaFold predicted structures, we extracted several features including Euclidean distances of the top *k* neighbors for each residue, the vectors starting from the carbon alpha (hereafter referred to as Cα) of a source residue to the Cα of a destination residue (edge vectors), the vectors of the same type of atoms from the forward and backward adjacent residues (orientation vectors), the unit vectors from nitrogen (hereafter referred as N) to Cα and from Cα to carbon (hereafter referred as C) (side-chain vectors), and the predicted local-distance difference test (pLDDT) accuracy of each Cα predicted by AlphaFold. These features are referred to as structure-based features and are input into the GVP layers.

To avoid the inaccurately predicted residues that may affect our prediction, we not only masked the 3D coordinates of the residues with pLDDT scores less than 0.9 but also combined the embeddings of pLDDT scores with structure-based features in GVP layers. We tested a range of pLDDT score thresholds from 0.5 to 1 with a 0.1 internal. The pLDDT threshold of 0.9 removed approximately 53.52% of residues from the training and validation data. The deep learning model trained with a 0.9 threshold outperformed in terms of validation loss (Supplementary Table S1).

The features, such as orientation vectors and side-chain vectors, have directions and are therefore considered vector features, whereas others like Euclidean distances and pLDDTs are scalar features. To unify the dimensions of channels, we embedded these features into a space of 128 dimensions, which are then input into the GVP layers.

##### Structural-rotation and dihedral features

We first discuss rotation-equivariant and rotation-invariance properties as these two concepts are important to understand why we apply a rotation on the query tertiary structure. Both rotation-equivariant and rotation-invariance features satisfy f(T(structure)) = T(f(strucure)), where T is a rotation function and f is a function of generating features. However, for rotation-equivariant features, f(T(strucure)) != f(strucure), which means the rotation causes the change of the feature values. Vector features, which contain directions, are rotation-equivariant features, such as edge vectors and orientation vectors.

Scalar features, on the other hand, are rotation-invariance, such as dihedrals and Euclidean distances, which satisfies f(T(strucure)) = f(strucure), meaning that the output remains the same even though the input is rotated.

A GVP layer is rotation-equivariant, but the biological functions of proteins are not affected by the rotations of protein structures. Therefore, for each input tertiary structure, we unify its orientation in the 3D space or get its local reference frame, which is defined based on the positions of N, Cα, and C atoms of each amino acid, using the implementation (36) of the algorithm proposed in (45).

Dihedral degrees are also used as input, which is defined as the angle between the two hyperplanes formed by the N, Cα and C atoms of adjacent residues.

##### Sequential features

The evolutionary-scale language modeling (ESM) (19) of the amino acid sequence of the query protein is also used as one input of the GVP layers. Furthermore, the token, which is a number encoded from each amino acid, is input into the summation function that adds the embeddings of the tokens, rotation-invariance features, dihedral degrees, ESM features, and pLDDTs. We set the embedding dimension of all of these features to 128, which makes the summation of these different features possible. We then pass the summation feature to the transformer layers, which will be discussed in a later section.

#### GVP layers

We feed the embeddings of Euclidean distances, edge vectors, orientation vectors, side-chain vectors, pLDDTs, and ESM into GVP layers (39). PANDA-3D has two GVP layers, which encode the sequential and structural features into intermediate features. As depicted in Figure 1B, a GVP layer has scalar and vector channels, where rotation-invariant scalar features $S_n\in \mathbb {R}^n$ and rotation-equivariant vector features $V_v\in \ \mathbb {R}^{v\times 3}$ are fed, respectively.


(1) \begin{equation*} V_h=Linear\left(V_v\right) . \end{equation*}

The vector features *V*_$v$_ are updated by a linear layer to $V_h\ \in \ \mathbb {R}^{h\times 3}$, where h is max ($v$, *n*).


(2) \begin{equation*} S_h=||V_h||_2 . \end{equation*}

We apply a *L*_2_ norm to transform the vector features *V*_*h*_ into a scalar feature *S*_*h*_.


(3) \begin{equation*} S_m=Linear(concat(S_h,S_n)) . \end{equation*}

The scalar features *S*_*h*_ and *S*_*n*_ are concatenated and then updated to *S*_*m*_ by a linear layer.


(4) \begin{equation*} V_\mu =Linear\left(V_h\right) . \end{equation*}

Another linear layer is then applied to update *V*_*h*_ to *V*_μ_.


(5) \begin{equation*} v_\mu =||V_\mu ||_2 . \end{equation*}

(6) \begin{equation*} V^\prime = \sigma ^+(v_\mu )\bigodot \ V_\mu . \end{equation*}

One of the final outputs of the GVP layers *V*′ is calculated by applying a nonlinear scale function σ^+^ on the normalized $v$_μ_, followed by a row-wise multiplication of *V*_μ_.


(7) \begin{equation*} S^\prime = \sigma (S_m) . \end{equation*}

The other output of the GVP layers *S*′ is calculated by applying a nonlinear scale function σ on *S*_*m*_.

#### Transformer layers

As depicted in Figure 1A and C, we applied the decoder layers (44) that are implemented differently from the original transformer architecture (40). The original architecture (40) is for generating sentences or sequences of words on machine translation tasks with masking during training, but our task is for multi-label classifications without using attention masks. Each decoder layer has a multi-head self-attention layer, a multi-head cross-attention layer, and a linear feed-forward layer. In the decoder self-attention layer, all of the keys, values and queries are the embedding of candidate GO terms.

A decoder multi-head cross-attention layer calculates attention over the keys and values from the encoder output and the query from the previous self-attention layer. After that, a linear layer is added followed by a sigmoid function that generates the final outputs, which can be considered as the confidence scores for each candidate GO term.

### Implementation and training details

We implemented PANDA-3D based on the code from the PyTorch library (46), ESM inverse folding (36), GVP (39) and Transformer (40). The model has eight million trainable parameters. We parallelly trained the model on two NVIDIA A100 GPUs with 40GB of memory each. The model converged after about 18 hours of training. We utilized a binary cross-entropy loss function with a logarithm function for training. To account for the significant difference between the numbers of negatively labeled GO terms and positively labeled GO terms in our training data set, we assign a weight of 3.0 to all of the positive classes when the loss value is computed. We used a batch size of eight, attention heads of eight, and a learning rate of 0.0001 with Adam optimizer. We conducted experiments with different numbers of GVP layers, numbers of decoder layers, learning rates, and batch sizes. The performance results are available in the Supplementary Tables S2 and S3.

### Computational time and scalability

Our benchmark showed that the runtime of PANDA-3D for a protein that has 1093 residues on a Tesla V100 was 36 seconds (not including the time for generating the AlphaFold model), and GPU memory usage was about 1 GB. A limitation of PANDA-3D is that it needs the AlphaFold model as input, but AlphaFold DB includes the models for more than 200 million proteins (47). For the cases that a model is not included in AlphaFold DB, a user can generate an AlphaFold model using DeepMind’s Colab notebook or open-source code at https://alphafold.ebi.ac.uk/ (45,47).

### Datasets

We downloaded the manually-reviewed protein sequences and experimentally determined (with the evidence codes: EXP, IDA, IMP, IGI, IEP, TAS or IC) protein functions in the format of GO terms from Swiss-Prot (48), which were released on 25 May 2022. All three ontologies of GO terms were used including molecular function ontology (MFO), biological process ontology (BPO), and cellular component ontology (CCO). The downloaded GO terms are nodes in the GO-directed acyclic diagrams (DAGs). For training, we propagated the nodes up to the root node, and all of the nodes and their ancestors are considered positively labeled GO terms for each protein. We used the GO definition released on 1 July 2022 (49). We downloaded all of the predicted protein tertiary structures as of May 2022 from AlphaFold DB (45,47).

A total of 68,523 proteins have both experimentally determined GO terms and AlphaFold models. We randomly split the proteins into training (80%), validation (10%), and testing (10%). Protein sequences from the testing set were searched against the training dataset of PANDA-3D and the other tools that we compared with, and those with a maximum PSI-BLAST (13) identity score greater than a cut-off value were removed. This is to make sure that the blind test data set has no overlap with the training datasets for a fair comparison of the performance. We tested the performance with different identity score cut-off values shown in a later section.

We only used the GO terms that had been annotated with at least 50 proteins in the training dataset as the candidate GO terms or machine-learning target classes. This is to ensure that the GO terms that PANDA-3D can predict have enough training data and good accuracy, but a tradeoff of this is that it reduces the number of GO terms that PANDA-3D can predict. We benchmarked two different numbers of proteins (45 and 50) for a candidate GO term to be included, and 50 resulted in slightly better performance (data not shown). The number of proteins in the training, validation, and testing datasets and the number of GO terms or machine-learning classes are shown in Table 1.

### Evaluation metrics

We evaluated the methods using both protein- and term-centric evaluation measures, which were what the official assessment measures used in CAFA2, CAFA3 and CAFA-π (7,11). Both measures were performed on propagated predictions and propagated ground truths. In propagated predictions, a GO term’s confidence score was updated to the highest predicted score among its descendant GO terms. All of the ancestor GO terms of the downloaded leaf GO terms are considered positively annotated. We performed the maximum *F*-measure (*F*_*max*_), minimum semantic distance (*S*_*min*_), and area under the precision-recall curve (AUPR) for the protein-centric evaluations, and used the area under the receiver operating characteristic (ROC) curve (AUROC) metric for the GO-centric evaluation as performed in CAFA (7,11,50).

The *F*_*max*_ is calculated over a set of confidence-score thresholds as:


(8) \begin{equation*} F_{max}=max\left\lbrace \frac{2\times p r(t)\times r c(t)}{pr(t)+rc(t)}\right\rbrace , \end{equation*}

where *pr*(*t*) and *rc*(*t*) are precision and recall for all testing proteins over a threshold *t*, respectively. They are calculated as:


(9) \begin{equation*} pr(t) = \frac{1}{m(t)}\times \sum _{i=1}^{m(t)}{{pr}_i(t)}, \end{equation*}

(10) \begin{equation*} rc(t) = \frac{1}{n}\times \sum _{i=1}^{n}{{rc}_i(t)}, \end{equation*}

where *m*(*t*) represents the number of proteins with at least one GO term having a confidence score greater than or equal to *t*, and *n* denotes the number of total testing proteins. *pr*_*i*_(*t*) and *rc*_*i*_(*t*) are precision and recall of a protein that was calculated as:


(11) \begin{equation*} {pr}_i\left(t\right) = \frac{\sum _{f}{I(f\ \epsilon \ P_i}\wedge \ f\ \epsilon \ T_i)}{\sum _{f}{I(f\ \epsilon \ P_i})}, \end{equation*}

(12) \begin{equation*} {rc}_i\left(t\right)= \frac{\sum _{f}{I(f\ \epsilon \ P_i}\wedge \ f\ \epsilon \ T_i)}{\sum _{f}{I(f\ \epsilon \ T_i})}, \end{equation*}

where *P*_*i*_ represents the predicted GO term set having confidence scores greater than or equal to *t*, *T*_*i*_ presents the true GO term set, *f* denotes a GO term, and *I* represents an indicator function. We calculated the precision and recall for a protein when *P*_*i*_ contained at least one GO term when a maximum identity cut-off value was applied.

The calculation of *S*_*min*_ takes into account the unbalanced information content (IC) of GO terms. The information content (IC) of a GO term is calculated on all of the 68,523 proteins as follows:


(13) \begin{equation*} IC(f) = {-log}_{10}\frac{{Occur}_f}{{Occur}_{all_terms}}, \end{equation*}

where *f* is a GO term, *Occur*_*f*_ indicates the occurrences of *f* and its descendants, and ${Occur}_{all_terms}$ is the total occurrence of all GO terms.

The *S*_*min*_ is calculated as:


(14) \begin{equation*} S_{min}={_t^{min}}\left\lbrace \sqrt{ru\left(t\right)^2+mi\left(t\right)^2}\right\rbrace , \end{equation*}

where *ru*(*t*) and *mi*(*t*) are the average of remaining uncertainty *ru*_*i*_(*t*) and misinformation *mi*_*i*_(*t*), respectively. These measures are calculated using the following formulas:


(15) \begin{equation*} ru\left(t\right) = \frac{1}{n}\times \sum _{i=1}^{n}{{ru}_i\left(t\right)}, \end{equation*}

(16) \begin{equation*} mi\left(t\right) = \frac{1}{n}\times \sum _{i=1}^{m}{{mi}_i\left(t\right)}, \end{equation*}

(17) \begin{equation*} {ru}_i\left(t\right) = \sum _{f}{IC(f)\times \ I(f\notin \ P_i(t)}\wedge \ f\in \ T_i, \end{equation*}

(18) \begin{equation*} {mi}_i\left(t\right) = \sum _{f}{IC(f)\times \ I(f\in \ P_i(t)}\wedge \ f\notin \ T_i, \end{equation*}

where *i* means the measure of a protein, *f* represents a GO term, and *I* represents an indicator function.

## Results

### Overview

We performed both protein- and term-centric evaluations on the testing dataset. We compared PANDA-3D to a similar method, DeepFRI, which also utilizes tertiary structures and sequences for protein function prediction. We used AlphaFold-predicted structures instead of experimentally determined structures in our testing. We were unable to compare our approach with another similar method, GAT-GO, as neither its training data nor trained model is available.

We used two methods as baselines: Naïve and BLAST. The Naïve baseline method predicts GO terms based on the relative frequency of each GO term in the Uniprot Swiss Prot database. The BLAST method predicts GO terms by transferring the experimental GO terms of similar sequences found in the training dataset using PSI-BLAST (51), where the predicted scores are the maximum identity scores.

On the same dataset as PANDA-3D, we also trained and evaluated DeepGOCNN that is the neural network component of DeepGOPlus, which is a CNN-based network designed to directly predict protein functions from amino acid sequences (14).

On the second and third testing datasets, we performed protein-centric evaluations to compare PANDA-3D with DeepGO-SE and PANDA-3D with SPROF-GO, respectively. The details about the methodologies and/or training of the baseline methods, DeepGOCNN, DeepFRI, DeepGO-SE, and SPROF-GO can be found in the supplementary materials.

### The performance of PANDA-3D on *F*_*max*_, *S*_*min*_ and AUPR

**Figure 2.**
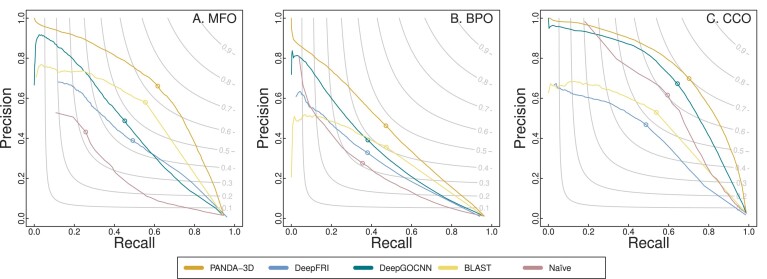
The *F*_*max*_ scores and precision-recall curves of PANDA-3D, DeepFRI, DeepGOCNN, Naïve and BLAST. The benchmark was performed on the testing dataset labeled as ‘DeepFRI’ in Table 1 with the maximum sequence identity cutoff of 0.95.

Figure 2 shows the precision-recall curves and *F*_*max*_ scores for comparing PANDA-3D, DeepFRI, DeepGOCNN, Naïve and BLAST, which indicates that PANDA-3D outperforms DeepFRI and all baseline methods. Table 2 shows the performance of these methods in terms of *F*_*max*_, *S*_*min*_ and AUPR on the first testing dataset labeled as ‘DeepFRI’ in Table 1 with the maximum sequence identity cutoff of 0.95. PANDA-3D achieved the best *F*_*max*_ scores for all three GO categories: 0.642 for MFO, 0.471 for BPO, and 0.705 for CCO. PANDA-3D also outperforms all other methods in terms of *S*_*min*_ and AUPR.

**Table 1.** The number of proteins in the training, validation and testing datasets and the number of GO terms or machine-learning classes. The sequences in three separate testing datasets were searched against the training datasets of PANDA-3D and DeepFRI, PANDA-3D and DeepGO-SE, and PANDA-3D and SPROF-GO, respectively. The testing sequences with a maximum sequence identity score cutoff greater than a cut-off value were removed

Dataset		# of proteins
Training		51 245
Validation		6419
Testing (DeepFRI) with identity score cutoff of	0.95	4719
	0.8	3533
	0.7	3024
	0.6	2506
	0.5	1917
	0.4	1167
Testing (DeepGO-SE) with identity score cutoff of	0.95	260
	0.8	198
	0.7	176
	0.6	154
	0.5	127
	0.4	91
Testing (SPROF-GO) with identity score cutoff of	0.95	461
	0.8	351
	0.7	282
	0.6	234
	0.5	183
	0.4	117
Ontologies		# of GO terms
MFO classes		438
BPO classes		3105
CCO classes		405

**Table 2.** The performances of PANDA-3D, DeepFRI, DeepGOCNN, Naïve and BLAST for *F*_max_, *S*_min_ and AUPR. The highest *F*_max_, the smallest *S*_min_, and the highest AUPR are in bold and italics. The benchmark was performed on the testing dataset labeled as ‘DeepFRI’ in Table 1

	*F*_max_			*S*_min_			AUPR		
Method	MFO	BPO	CCO	MFO	BPO	CCO	MFO	BPO	CCO
Naïve	0.322	0.311	0.605	11.381	49.404	12.728	0.197	0.227	0.525
BLAST	0.567	0.407	0.534	9.269	49.211	12.566	0.498	0.305	0.465
DeepGOCNN	0.469	0.387	0.658	9.834	46.861	11.635	0.444	0.336	0.69
DeepFRI	0.435	0.352	0.477	9.894	48.079	12.56	0.305	0.257	0.377
PANDA-3D	***0.642***	***0.471***	***0.705***	***7.29***	***43.601***	***10.027***	***0.654***	***0.445***	***0.766***

**Figure 3.**
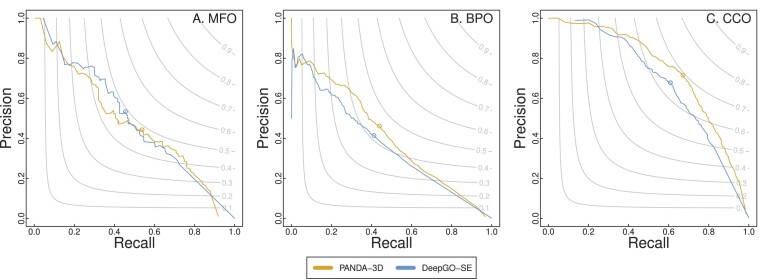
The *F*_*max*_ scores and precision-recall curves of PANDA-3D and DeepGO-SE. The benchmark was performed on the testing dataset labeled as ‘DeepGO-SE’ in Table 1 with the maximum sequence identity cutoff of 0.95.

**Figure 4.**
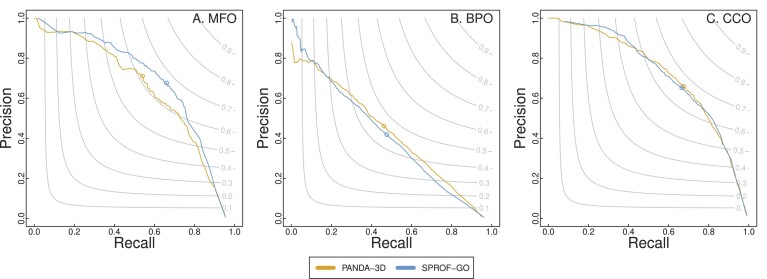
The *F*_*max*_ scores and precision-recall curves of PANDA-3D and SPROF-GO. The benchmark was performed on the testing dataset labeled as ‘SPROF-GO’ in Table 1 with the maximum sequence identity cutoff of 0.95.

Figure 3 presents the precision-recall curves and *F*_*max*_ scores for comparing PANDA-3D with DeepGO-SE. PANDA-3D outperforms DeepGO-SE for BPO and CCO almost all the time while exhibiting comparable performance in terms of MFO. In Figure 4, the precision-recall curves and *F*_*max*_ scores of PANDA-3D and SPROF-GO show that PANDA-3D outperforms or is comparable to SPROF-GO for BPO and CCO but slightly worse in MFO.

Table 3 shows the performance of PANDA-3D and DeepGO-SE in terms of *F*_*max*_, *S*_*min*_ and AUPR on the second testing dataset labeled as ‘DeepGO-SE’ in Table 1 with the maximum sequence identity cutoff of 0.95. PANDA-3D outperformed DeepGO-SE in eight out of nine scores. Table 4 shows that PANDA-3D outperformed SPROF-GO in BPO and CCO, securing six out of nine scores on the third testing dataset labeled as ‘SPROF-GO’ in Table 1 with the maximum sequence identity cutoff of 0.95.

**Table 3.** The performances of PANDA-3D and DeepGO-SE for *F*_max_, *S*_min_ and AUPR. The highest *F*_max_, the smallest *S*_min_, and the highest AUPR are in bold and italics. The benchmark was performed on the testing dataset labeled as ‘DeepGO-SE’ in Table 1 with the maximum sequence identity cutoff of 0.95

	*F*_max_			*S*_min_			AUPR		
Method	MFO	BPO	CCO	MFO	BPO	CCO	MFO	BPO	CCO
DeepGO-SE	***0.491***	0.413	0.641	7.665	30.897	9.925	0.447	0.368	0.554
PANDA-3D	0.486	***0.45***	***0.692***	***7.526***	***29.306***	***8.837***	***0.47***	***0.409***	***0.74***

**Table 4.** The performances of PANDA-3D and SPROF-GO for *F*_max_, *S*_min_ and AUPR. The highest *F*_max_, the smallest *S*_min_, and the highest AUPR are in bold and italics. The benchmark was performed on the testing dataset labeled as ‘SPROF-GO’ in Table 1 with the maximum sequence identity cutoff of 0.95

	*F*_max_			*S*_min_			AUPR		
Method	MFO	BPO	CCO	MFO	BPO	CCO	MFO	BPO	CCO
SPROF-GO	***0.669***	0.446	0.659	***5.47***	31.84	8.432	***0.669***	0.409	0.642
PANDA-3D	0.614	***0.462***	***0.668***	5.69	***31.232***	***7.821***	0.641	***0.422***	***0.72***

**Figure 5.**
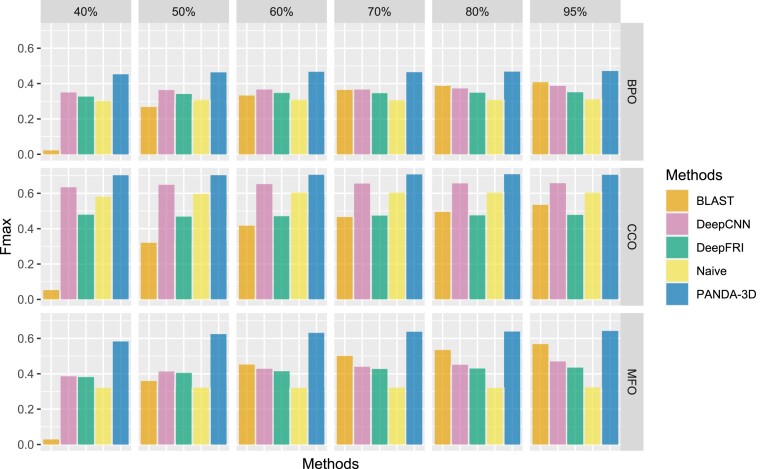
The Fmax scores of PANDA-3D, DeepFRI, DeepGOCNN, Naïve and BLAST at different maximum sequence identity cutoffs. 40%, 50%, 60%, 70%, 80% and 95% are maximum sequence identity cutoffs. Sequences from the testing set having a maximum identity score greater than the maximum sequence identity were removed. The benchmark was performed on the testing dataset labeled as ‘DeepFRI’ in Table 1.

**Figure 6.**
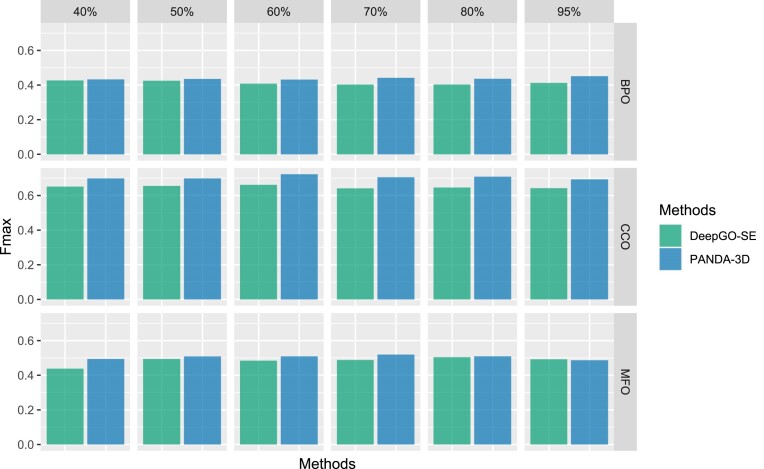
The Fmax scores of PANDA-3D and DeepGO-SE at different maximum sequence identity cutoffs. 40%, 50%, 60%, 70%, 80% and 95% are maximum sequence identity cutoffs. Sequences from the testing set having a maximum identity score greater than the maximum sequence identity were removed. The benchmark was performed on the testing dataset labeled as ‘DeepGO-SE’ in Table 1.

**Figure 7.**
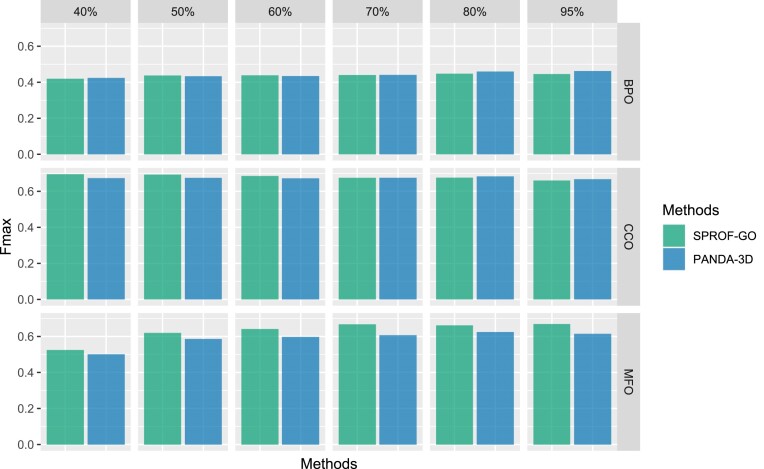
The Fmax scores of PANDA-3D and SPROF-GO at different maximum sequence identity cutoffs. 40%, 50%, 60%, 70%, 80% and 95% are maximum sequence identity cutoffs. Sequences from the testing set having a maximum identity score greater than the maximum sequence identity were removed. The benchmark was performed on the testing dataset labeled as ‘SPROF-GO’ in Table 1.

Figure 5 displays the *F*_*max*_ scores of PANDA-3D and other methods with different maximum identity cutoffs. The performance of BLAST improves as the cutoff value increases, while the performance of other methods is not significantly affected by cutoffs. PANDA-3D outperforms all other methods on *F*_*max*_ at different cutoffs. Figure 6 plots *F*_*max*_ scores of PANDA-3D and DeepGO-SE with different maximum sequence identity cutoffs. PANDA-3D almost always outperformed DeepGO-SE. Figure 7 plots the *F*_*max*_ scores of PANDA-3D and SPROF-GO with different maximum sequence identity cutoffs. PANDA-3D outperformed SPROF-GO at 80% and 95% cutoffs and showed similar performance at other cutoffs for BPO and CCO, while SPROF-GO outperforms PANDA-3D for MFO.

### Feature importance

To interpret the significance of the features used in PANDA-3D, we permutated one feature every time to test its contributions towards accuracy. Specifically, we shuffled the values of each feature randomly at the residue level and examined the resulting changes in performance. The features we tested were: ESMs, tokens of sequence, dihedral, Euclidean distances of the top *k* neighbors, edge vectors, orientation vectors, and pLDDTs. The results shown in Table 5 suggest that ESM, token, and dihedral angle are the top three important features, as shuffling these features results in the worst performance of PANDA-3D in terms of *F*_*max*_, *S*_*min*_ and AUPR.

**Table 5.** The performance of PANDA-3D with permutated features. The top three lowest *F*_*max*_, highest *S*_*min*_, and the lowest AUPR are in bold and italics. The benchmark was performed on the testing dataset labeled as ‘DeepFRI’ in Table 1 with the maximum sequence identity cutoff of 0.95

	*F*_max_			*S*_min_			AUPR		
Permutated Features	MFO	BPO	CCO	MFO	BPO	CCO	MFO	BPO	CCO
None	0.642	0.471	0.705	7.29	43.601	10.027	0.654	0.445	0.766
ESM	***0.63***	***0.458***	***0.7***	***7.539***	***44.249***	***10.218***	***0.64***	***0.431***	***0.761***
Token	***0.634***	***0.465***	***0.7***	***7.421***	***43.934***	***10.151***	***0.647***	***0.437***	***0.763***
Dihedral	***0.631***	***0.463***	***0.701***	***7.457***	***44.024***	***10.177***	***0.643***	***0.435***	***0.762***
Euclidean distance	0.642	0.471	0.705	7.29	43.605	10.028	0.654	0.445	0.766
Edge vector	0.642	0.471	0.705	7.291	43.601	10.026	0.654	0.445	0.766
Orientation vector	0.641	0.47	0.704	7.295	43.627	10.023	0.651	0.443	0.764
pLDDT	0.643	0.471	0.704	7.286	43.58	10.034	0.654	0.445	0.765
Side-chain vector	0.642	0.47	0.705	7.291	43.611	10.034	0.654	0.444	0.765

### The performance of PANDA-3D on term-centric evaluation

We report the term-centric evaluation results in Table 6, which shows the AUROCs of the methods based on all of the candidate GO terms in PANDA-3D and another two individual GO terms: biofilm formation (GO:0042710) and motility (GO:0001539). The 0.5 AUROCs of Naïve is caused by the design of the Naïve predictor (see supplementary materials) making it predict the same confidence scores for all of the candidate GO terms for all of the proteins in the testing dataset.

**Table 6.** Term-centric evaluations of PANDA-3D, DeepFRI, DeepGOCNN, Naïve and BLAST, in which the AUROCs on all candidate GO terms, biofilm formation (GO:0042710) and motility (GO:0001539) are reported. The benchmark was performed on the testing dataset labeled as ‘DeepFRI’ in Table 1 with the maximum sequence identity cutoff of 0.95

Methods	All GO-term classes	GO:0042710	GO:0001539
PANDA-3D	0.897924	0.942165	0.872102
DeepFRI	0.530414	0.418858	0.342771
BLAST	0.665574	0.525296	0.581225
Naïve	0.5	0.5	0.5
DeepGOCNN	0.809302	0.766282	0.721743

This type of evaluation was also used in CAFA2, CAFA3 and CAFA-π to rank predictors. In the CAFA3 and CAFA-π evaluations, three GO terms were used for evaluation, but among these three GO terms, only two GO terms: biofilm formation (GO:0042710) and motility (GO:0001539), are the candidate GO terms of PANDA-3D. Therefore, we only evaluated the performances of PANDA-3D on these two GO terms. Predicting these two GO terms is challenging in CAFA3 and CAFA-π since the best AUROC of the top five teams when predicting GO:0042710 only slightly exceeds the BLAST baseline (7).

PANDA-3D outperforms the BLAST and other methods significantly when predicting all candidate GO terms, biofilm formation (GO:0042710), and motility (GO:0001539).

## Discussion and conclusions

The successful performance of AlphaFold in predicting protein tertiary structures allows the community to have access to a large number of models with good qualities. We developed PANDA-3D, a novel method that predicts protein functions from AlphaFold models and protein sequences. PANDA-3D combines GVP-GNN layers and decoder transformer layers that capture the 3D features from the AlphaFold models and then predict GO terms. PANDA-3D was evaluated for protein function prediction using both protein- and term-centric evaluations and achieved noticeably better performances than an existing method that takes experimentally determined structures as input and other methods that take protein sequence as input. Permutation feature importance analysis revealed that ESMs, tokens of sequence, and dihedrals are the top three most important features. In term-centric evaluation, PANDA-3D significantly outperformed other methods in predicting biofilm formation (GO:0042710), motility (GO:0001539), and all GO-term classes, which suggests that PANDA-3D is a highly accurate method compared to the top methods in CAFA3 and CAFA-π.

PANDA-3D for accurately predicting protein functions can speed up the search for molecular compounds with the potential to cure a disease by reducing the number of clinical candidate molecules, which is a process that can take years. The alteration of biological activity between proteins and drugs is primarily determined by their structures. Unlike traditional protein function predictors that predict protein functions from sequences, PANDA-3D predicts protein functions based on protein 3D structures. As the predicted structures become more accurate, the advantages of structure-based protein function prediction will be further obvious.

A limitation of PANDA-3D is its reliance on AlphaFold models as input. If a model is not in the AlphaFold DB, users must generate the AlphaFold model first.

## Supplementary Material

lqae094_Supplemental_File

## Data Availability

The web server of PANDA-3D can be freely accessed from http://dna.cs.miami.edu/PANDA-3D. The source code, training, validation, and testing datasets and trained models of PANDA-3D can be found at http://dna.cs.miami.edu/PANDA-3D and https://github.com/zwang-bioinformatics/PANDA-3D.
