# Supplementary material for: PANDA-3D: protein function prediction based on AlphaFold models
Source: NAR Genom Bioinform. 2024 Aug 6;6(3):lqae094. doi: 10.1093/nargab/lqae094 (PMC11302463; doi:10.1093/nargab/lqae094)
Supplement: lqae094_Supplemental_File [file lqae094_supplemental_file.pdf]

PANDA-3D: protein function prediction based on AlphaFold models

Chenguang Zhao[1], Tong Liu[2], and Zheng Wang[2,*]

[1]Computer and Information Sciences Department, St. Ambrose University, 518 W Locust St, Davenport, 52803, IA, USA

[2]Department of Computer Science, University of Miami, 1365 Memorial Drive, Coral Gables, FL, 33124, USA

*To whom correspondence should be addressed.

Contact: zheng.wang@miami.edu

1. Methods used for comparison

1.1 Naïve baseline

The Naïve method predicts GO terms based on the relative frequency of GO terms in the Uniprot Swiss Prot database. This involved three steps: calculating the occurrences of GO terms in the database; applying a logarithm base 2 to those occurrences; and normalizing all occurrences by dividing them by the maximum occurrence.

1.2 BLAST baseline

The BLAST method predicts GO terms by transferring experimental GO terms from similar sequences identified in the training dataset using PSI-BLAST. Predicted scores are determined as the maximum identity scores. PSI-BLAST conducts iterative searches against the training dataset until either the maximum number of iterations is reached or no new sequences are found. We set the maximum number of iterations as two. Only search results with an e-value below 0.05 are used for prediction.

1.3 DeepGOCNN

DeepGOCNN was trained and benchmarked on the same datasets as PANDA-3D. DeepGOCNN model was implemented following the architecture described in the paper by (Kulmanov and Hoehndorf, 2020). The model takes one-hot encoded amino acid sequences as input. It utilizes one-dimensional convolutional layers with various kernel sizes (8, 16, 24, …,128) applied to the one-hot encoded data with 21 input channels and 512 output channels. Following the convolutional layers,

max-pooling layers were applied. The outputs from the max-pooling layers were flattened and concatenated to form a 1x8192 vector. This was followed by a fully connected layer, projecting the output to the number of classes. Weight initialization was carried out using Xavier initialization. A learning rate of 0.0003 and a batch size of 32 were used for training.

1.4 DeepFRI

The pre-trained DeepFRI model was downloaded from https://github.com/flatironinstitute/DeepFRI. We employed the pre-trained models to make predictions on the testing dataset labeled as ”DeepFRI” in Table 1. Sequences from the testing dataset were searched against the training datasets of PANDA-3D and DeepFRI, and any sequences with a maximum PSI-BLAST identity score greater than a specified cutoff value were excluded.

1.5 DeepGO-SE

The prediction of DeepGO-SE was provided by the authors upon request. To create the testing dataset labeled as ”DeepGO-SE” in Table 1, we downloaded the training dataset of DeepGO-SE from https://github.com/bio-ontology-research-group/deepgo2. Sequences from the testing dataset were searched against the training datasets of PANDA-3D and DeepGO-SE, and any sequences with a maximum PSI-BLAST identity score greater than a specified cutoff value were excluded.

1.6 SPROF-GO

The prediction of SPROF-GO was provided by the authors upon request. We downloaded the training dataset of SPROF-GO from https://github.com/biomed-AI/SPROF-GO/tree/main/datasets. We then searched the sequences from the testing dataset against the training datasets of PANDA-3D and SPROF-GO, excluding any sequences with a maximum PSI-BLAST identity score greater than a specified cutoff value. The testing dataset was labeled as ”SPROF-GO” in Table 1.

2. Performances of PANDA-3D in additional experiments

Table S1 The validation loss of PANDA-3D using different pLDDT score thresholds.

pLDDT score thresholds	Validation loss
0.5	0.076
0.6	0.076
0.7	0.075
0.8	0.075
0.9	0.075
1.0	0.076

Table S2 PANDA-3D architecture performances as the number of GVP layers and decoder layers changes. PANDA-3D used two GVP layers and two decoder layers.

# of GVP layer	# of Decoder layer	Validation loss
2	1	0.076939
2	2	0.075992
2	3	0.076809
1	2	0.075725
3	2	0.075962
4	2	0.076213

Table S3 PANDA-3D architecture performances as the learning rate and batch size changes. PANDA-3D used a learning rate of 0.0001 and a batch size of eight.

Learning rate	Batch size	Validation loss
0.0001	8	0.075992
0.0001	6	0.07599
0.0001	4	0.075851
0.001	8	0.106433
0.00001	8	0.080616

References

Kulmanov, M. and Hoehndorf, R. DeepGOPlus: improved protein function prediction from sequence. *Bioinformatics* 2020;36(2):422-429.
